# Point prevalence survey of antibiotic allergies in hospitalized patients

**DOI:** 10.1017/ash.2025.10165

**Published:** 2025-10-17

**Authors:** Simo Sirkeoja, Hanna Viskari, Jaana Syrjänen, Reetta Huttunen, Meeri Honkanen

**Affiliations:** 1 Infectious Disease Unit, Department of Internal Medicine, Tampere University Hospital, Wellbeing Services County of Pirkanmaa, Tampere, Finland; 2 Faculty of Medicine and Health Technology, Tampere University, Tampere, Finland

## Abstract

As part of healthcare-associated infections prevalence survey, we examined the prevalence of antibiotic allergies among inpatients and the possibility to de-label reported penicillin and sulfa allergies. Results show that most of the patients with a penicillin or sulfa allergy label are eligible for either direct de-labeling or oral antibiotic challenge.

## Introduction

Reported antibiotic allergies significantly limit the use of first-line antibiotics. Studies have shown the harmful effects of antibiotic allergies on treatment outcomes.^1–2^ Furthermore the evaluation of antibiotic allergies has been acknowledged as an important tool in antimicrobial stewardship (AMS).^3^

Penicillin is the most common cause of reported drug allergy, followed by sulfa. However, true penicillin allergy is rare and grossly overestimated.^2^ Data on sulfa allergy evaluation is more limited, but it is likely that many of them could also be de-labeled.^4^ The prevalence of reported allergies to other antibiotic groups is low, but less studied.^3,5^

Guidelines encourage to re-evaluate the correctness of antibiotic allergy labels.^2,3^ Direct oral challenge has been found to be safe for low-risk penicillin allergy patients.^3^ The PEN-FAST score (including severity and time since reaction, and whether treatment was required) facilitates the assessment of penicillin allergies.^6^ An algorithm (SULF-FAST) using similar parameters for evaluating sulfa allergy has also been developed.^7^

The aim of this study was to examine the prevalence of antibiotic allergies among inpatients and to assess the possibility to de-label reported penicillin and sulfa allergies.

## Material and methods

This point-prevalence study was performed in the Tampere University Hospital, Finland. As part of the routine healthcare-associated infections’ control measures, a prevalence survey is carried out biannually. In the survey, infections and use of antibiotics of the hospitalized patients are recorded. Information on antibiotic allergies was included in 2024.

The study covered all patients who were hospitalized during the prevalence survey periods in March and September 2024. All recorded antibiotic allergies of the study patients were identified. The researcher (S.S.) checked the electronic health records (EHR) of the patients with a recorded antibiotic allergy (*n* = 170) and collected more information about the type of allergic reactions, the time since the reaction and the quality of the documentation. If the data was incomplete, the researcher (S.S.) personally interviewed the patients. Previous antibiotic treatments were also checked from the EHRs.

Reported allergies were roughly categorized into two types: nonallergic reactions (eg, antibiotic associated diarrhea) and hypersensitivity reactions (HSRs). HSR’s were subcategorized based on the timing of symptom onset.

In addition, for patients with a label of penicillin or sulfa allergy the appropriateness of the allergy label was evaluated. If the patient had been re-exposed without symptoms after the allergy label was recorded or if the reaction was evaluated as nonallergic, the patients were deemed eligible for direct de-labeling. For the remaining patients with a penicillin or sulfa allergy label, the PEN-FAST and SULF-FAST scores, respectively, were calculated. If the score was less than three points, the risk for a positive allergy test was considered low and the patient was evaluated to be eligible for a direct oral challenge.^6,7^

Differences between patients with and without an antibiotic allergy label were compared with χ2 test for categorical variables and Student’s t-test for continuous variables. A *p*-value < .05 was considered statistically significant. These analyses were performed using IBM SPSS Statistics (Version 26).

## Results

The study included 1,211 hospitalized patients. A total of 231 antibiotic allergy labels in 187 different patients (15% of the study patients) were recorded; 156 patients had an allergy to only one antibiotic. Patients with an antibiotic allergy label were older, more likely to be female and more likely to receive antibiotics (Table 1).

**Table 1.** Characteristics of the study population

	Total number of patients *n* = 1,211		Patients with antibiotic allergy *n* = 187		Patients without antibiotic allergy *n* = 1,024		*P*-value
**Age, mean (SD), y**	63.8 (24.2)		70.5 (16.3)		62.6 (25.2)		< .001
	N	%	N	%	N	%	
**Sex**							
Male Female	643 568	53.1 47.0	72 115	38.5 61.5	571 453	55.8 44.2	< .001
**Specialty**							
Medicine Surgery Pediatrics	731 396 84	60.4 32.7 6.9	119 64 4	63.6 34.2 2.1	612 332 80	59.8 32.4 7.8	.019
**Known MDRO carrier**							
MRSA ESBL Klebsiella CPE VRE	15 2 1 1	1.2 .2 .1 .1	2 0 0 0	1.1 0 0 0	13 2 1 1	1.3 .2 .1 .1	NA
**Antibiotic in use during study period**							
Yes No	337 874	27.8 72.2	63 124	33.7 66.3	272 752	26.6 73.4	.045

SD: standard deviation; MDRO: multidrug-resistant organism; MRSA: methicillin-resistant Staphylococcus aureus; ESBL: extended spectrum β-lactamase; CPE: carbapenemase-producing Enterobacterales; VRE: vancomycin-resistant Enterococcus; NA: not applicable.

Antibiotic allergy labels were recorded most frequently for penicillins (9%), followed by sulfa (3%), and cephalosporins (2%). Most of the allergic reactions had happened more than five years ago (199/231, 86 %). More recent allergic reactions were most commonly caused by cephalosporins. If the allergic reaction had occurred within five years, the type of reaction was recorded in 26/28 (93%) of cases, but in older allergies, the type of reaction was described in 128/199 (64%). (Supplementary Table)

**Figure 1.**
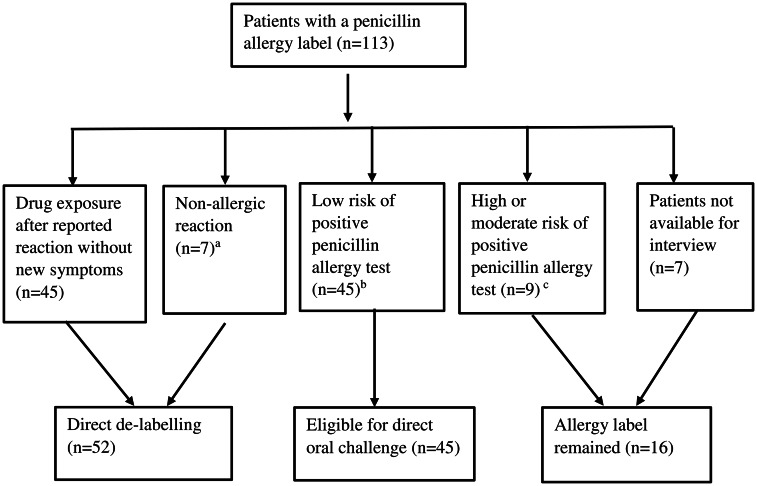
Evaluation of penicillin allergies. (a) Nonallergic reactions include diarrhea (2), nausea (3), fungal infection, leg pain. (b) Penicillin allergy clinical decision rule PEN-FAST score ≤ 2. (c) Penicillin allergy clinical decision rule PEN-FAST score ≥ 3.

**Figure 2.**
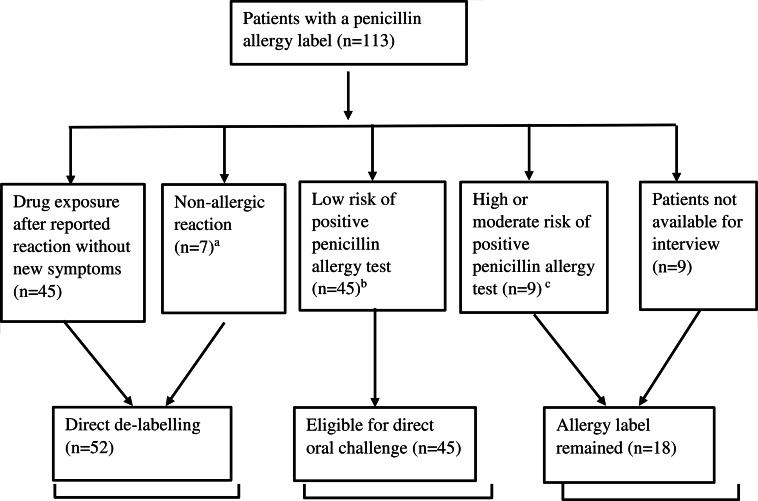
Evaluation of sulfa allergies. (a) Nonallergic reactions include diarrhea and an attack of restlessness. (B) sulfa allergy clinical decision rule SULF-FAST score ≤ 2. (c) Sulfa allergy clinical decision rule SULF-FAST score ≥ 3.

Results of penicillin allergy de-labeling are shown in figure 1: 52/113 (46%) were eligible for direct de-labeling based on patient history. For the remaining 61 patients, the PEN-FAST score was calculated: 74% (45/61) had a score less than 3, that is their allergy was classified as low-risk. Results of the sulfa allergy evaluation are shown in Figure 2.

## Discussion

The results of this study show that most of the hospitalized patients with a penicillin or sulfa allergy label would be eligible for oral antibiotic challenge or could be directly de-labeled.

In this study the prevalence of antibiotic allergies in hospitalized patients was 15%. It is in line with previously reported prevalences in hospitalized patients.^1,5^ Penicillin and sulfa were the most common culprits, as has been reported before.^1^ Interestingly cephalosporins were the most common causes of more recent allergic reactions. Previous studies have not reported any differences in antibiotic allergy labels in terms of when they have been recorded.

The recent guideline for the documentation of drug allergies in the EHRs highlights the importance of documenting reaction type, severity, and timing of symptoms.^8^ A point-prevalence study in long-term care facilities showed that up to 92.8% of the antibiotic allergy documentations were incomplete.^9^ In the current study two-thirds of the recorded allergies had a reaction type documented. Encouragingly, this was more likely for allergies reported within the last five years.

Interestingly, 40% of the patients with a penicillin allergy label had been given penicillin after the initial reaction, some of them inadvertently. Of those patients not eligible for direct de-labeling, three quarters were considered low-risk patients according to the PEN-FAST score. As NPV for true allergy in low-risk patients is almost 100%^10^ it can be estimated that up to 86% of the inpatients’ penicillin allergies could be de-labeled safely directly or by direct oral challenge.

Exposure to the drug after the initial reaction was much rarer for sulfa than for penicillin, highlighting the importance of direct drug challenge de-labeling patients with a sulfa allergy label. Various sulfa desensitization protocols have been recommended for patients with reported sulfa allergies, but recently direct oral challenge has been shown to be as effective and safe.^3^ The SULF-FAST score is a potential tool for finding suitable patients for direct oral challenge, but more studies are needed for it to be widely implemented.^7^

Checking the allergy labels as part of the routinely performed healthcare-associated infection prevalence survey does not add costs and takes less than a minute per patient. On the other hand, it gives an opportunity to educate healthcare professionals not only on infection control related issues, but also on antibiotic allergies. The results of the prevalence survey can be used as an aid in allergy de-labeling in the participating units.

A few limitations to this study must be acknowledged. First, not all patients could be contacted during their time in the hospital, so complete patient histories could not be collected. In addition, antibiotic challenges could not be made and so clinical outcomes remain unproven. Second, the study was conducted on hospital patients and cannot be fully generalized to outpatient care.

## Conclusions

In conclusion, this point-prevalence study shows that most of the hospitalized patients with a penicillin or sulfa allergy label would be eligible for either direct de-labeling or oral antibiotic challenge. We encourage other centers conducting healthcare-associated infection point-prevalence surveys to adopt this strategy of collecting antibiotic allergy data as part of their strategy on AMS.

## Supporting information

10.1017/ash.2025.10165.sm001 Sirkeoja et al. supplementary material Sirkeoja et al. supplementary material

## Supplementary material

The supplementary material for this article can be found at https://doi.org/10.1017/ash.2025.10165.
