# Supplementary material for: Point prevalence survey of antibiotic allergies in hospitalized patients
Source: Antimicrob Steward Healthc Epidemiol. 2025 Oct 17;5(1):e273. doi: 10.1017/ash.2025.10165 (PMC12538347; doi:10.1017/ash.2025.10165)
Supplement: Sirkeoja et al. supplementary material [file S2732494X25101654sup001.docx]

**Supplementary Appendix Table 1.** Number and characterization of reported antibiotic allergies

|  |  | Penicillins (n=113)^a^ | Cephalosporins (n=23)^b^ | Sulfonamides (n=42) | Fluoroquinolones (n=14)^c^ | Clindamycin (n=9) | Tetracyclines (n=9)^d^ | Metronidazole (n=4) | Trimethoprim (n=5) |
| --- | --- | --- | --- | --- | --- | --- | --- | --- | --- |
| Type and timing of the reaction | | | | | | | | | |
|  | HSR   - Immediate - Delayed - N/A   non-allergic  NA | 92  29  43  20  12  9 | 23  9  14  0  0  0 | 35  5  30  0  2  5 | 5  3  2  0  8  1 | 7  2  5  0  2  0 | 6  0  6  0  3  0 | 3  0  3  0  1  0 | 4  1  3  0  0  1 |
| Time since reaction | | | | | | | | | |
|  | <1 year  1–5 year  > 5 years  NA | 1  5  104  2 | 2  7  14  0 | 1  2  37  2 | 1  4  9  0 | 2  0  7  0 | 0  2  7  0 | 0  0  4  0 | 0  0  5  0 |
| Reaction type documented | | | | | | | | | |
|  | Yes  No | 70  43 | 22  1 | 24  18 | 11  3 | 8  1 | 7  2 | 3  1 | 4  1 |

HSR: hypersensitivity reaction; NA: not applicable

^a^ penicillin (n=92), amoxicillin (n=15), co-amoxiclav (n=4), piperacillin-tazobactam (n=2)

^b^ cephalexin (n=10), cefuroxime (n=12), ceftazidime (n=1)

^c^ levofloxacin (n=7), moxifloxacin (n=2), ciprofloxacin (n=5)

^d^ doxycycline (n=9)

Reported allergies not included in table 2 (n): erythromycin 3, neomycin 3, nitrofurantoin 3, chloramphenicol 1, pentamidine 1, vancomycin 1
